# Relationship between low-back pain and flexibility in children: A cross-sectional study

**DOI:** 10.1371/journal.pone.0293408

**Published:** 2023-11-10

**Authors:** Tadashi Ito, Hideshi Sugiura, Yuji Ito, Sho Narahara, Kentaro Natsume, Daiki Takahashi, Koji Noritake, Kazunori Yamazaki, Yoshihito Sakai, Nobuhiko Ochi

**Affiliations:** 1 Three-Dimensional Motion Analysis Laboratory, Aichi Prefectural Mikawa Aoitori Medical and Rehabilitation Center for Developmental Disabilities, Okazaki, Japan; 2 Department of Integrated Health Sciences, Graduate School of Medicine, Nagoya University, Nagoya, Japan; 3 Department of Pediatrics, Nagoya University Graduate School of Medicine, Nagoya, Japan; 4 Department of Pediatrics, Aichi Prefectural Mikawa Aoitori Medical and Rehabilitation Center for Developmental Disabilities, Okazaki, Japan; 5 Department of Orthopedic Surgery, Aichi Prefectural Mikawa Aoitori Medical and Rehabilitation Center for Developmental Disabilities, Okazaki, Japan; 6 Institutional Research Center, Aichi Mizuho College, Nagoya, Japan; 7 Department of Orthopedic Surgery, National Center for Geriatrics and Gerontology, Obu, Japan; Sohag University Faculty of Medicine, EGYPT

## Abstract

Low-back pain is common among school-aged children. Decreased trunk flexibility in childhood influences low-back pain in adulthood. Previous studies examining the association between low-back pain and trunk flexibility in children are insufficient. Examining this association among elementary school children may help to better understand trunk flexibility in children with low-back pain and to modify the management of inflexibility. Therefore, this study aimed to identify the prevalence of low-back pain and its relationship with physical function among elementary school students. School-aged children aged 6–12 years were recruited in Japan between May 2018 and March 2023. Fingertip-to-floor distance, back muscle strength, pelvic tilt angle during gait, and the visual analog scale for low-back pain were measured. In addition, factors independently related to low-back pain were determined through logistic regression analysis. Low-back pain was reported in 9.6% of the 394 participants (boys, 191; girls, 203). All children with low-back pain presented with back pain when they moved; however, the pain was non-specific. Logistic regression analysis showed that the fingertip-to-floor distance was an independent risk factor for low-back pain (odds ratio, 0.921; p = 0.007). The odds ratios calculated in the logistic regression analysis confirmed that low-back pain frequency increased as the fingertip-to-floor distance decreased. The risk of low-back pain was associated with inflexibility, regardless of sex and muscle strength. These findings suggest that children with low-back pain must increase their trunk and lower extremity flexibility.

## Introduction

Low-back pain is common in both children and adults [1]. Furthermore, back pain in school-aged children is related to the corresponding figure in adulthood [2]. Back pain prevalence increases with age, from 1% at age 7 to 6% at age 10 [3]. In a large survey of elementary and junior high school students in Japan, back pain was observed in 10.2% [4]. Therefore, low-back pain in this age group is likely a significant risk factor for developing low-back pain in adulthood [5, 6]. The risk factors and prevention strategies for low-back pain in children have not been studied in detail [7–9]. Moreover, the prevalence of back pain is considered to be increasing, especially between 11 and 12 years of age [10, 11]. A recent three-year study of spinal pain in 9-year-old elementary school children reported that both adolescent development and linear growth were associated with spinal pain [12]. Low-back pain in childhood is influenced by body mass index (BMI), physical activity, screen time, and sleep time; the clinical features are diverse [13, 14].

Recent studies have reported that trunk inflexibility affects low-back pain in adulthood [15, 16]. A relationship between low-back pain and hip flexibility has also been reported [17]. Lloyd et al. reported that children’s musculoskeletal structures are not adequately developed to support rapid changes in mechanical loading of the spine caused by the differential growth rates of the legs and trunk [18]. Hamstring inflexibility has been associated with low-back pain in adolescence and adulthood [19–21]; however, information is still lacking in studies of elementary school-aged children. Studies on muscle strength have demonstrated no association with low-back pain in healthy children 10–16 years of age [22]. Moreover, tight hamstring and quadriceps muscles have been reported to cause a posterior pelvic tilt in children and adolescents [23]. Children with low-back pain have shown significant improvements in hamstring flexibility with exercise and physical activity [24]. Other systematic reviews have demonstrated that there is a lack of understanding of the relationship between motor function levels, such as flexibility and muscle strength, and low-back pain, as well as the specifics by sex and age group [25]. Investigating the relationship between low-back pain and trunk flexibility in children will help provide new information about factors contributing to low-back pain in children. Based on these previous studies, it is hypothesized that children with back pain may be less flexible due to pain than those without back pain. Thus, understanding the relationship between the flexibility of trunk function and low-back pain may be helpful in the treatment of low-back pain in children. Furthermore, clarifying the association between the risk of low-back pain and flexibility may lead to the development of preventative strategies.

These findings highlight the importance of assessing spinal flexibility in children. Nevertheless, previous studies examining the association between low-back pain and trunk flexibility in children were insufficient. Recent reviews have concluded that the physical performance of the trunk and risk factors for low-back pain in children remain uncertain [10, 13]. Few studies have investigated the differences in flexibility among children in Japan with and without low-back pain by applying flexibility assessments. Additionally, no studies in Japan include flexibility, back strength, or pelvic tilt. Therefore, examining this association in elementary school children would help to better understand trunk flexibility in children with low-back pain and modify the management of inflexibility.

This study aimed to explore the association between low-back pain and flexibility in children and compare these parameters with those in the same age group without low-back pain.

## Materials and methods

### Study population

**Fig 1:**
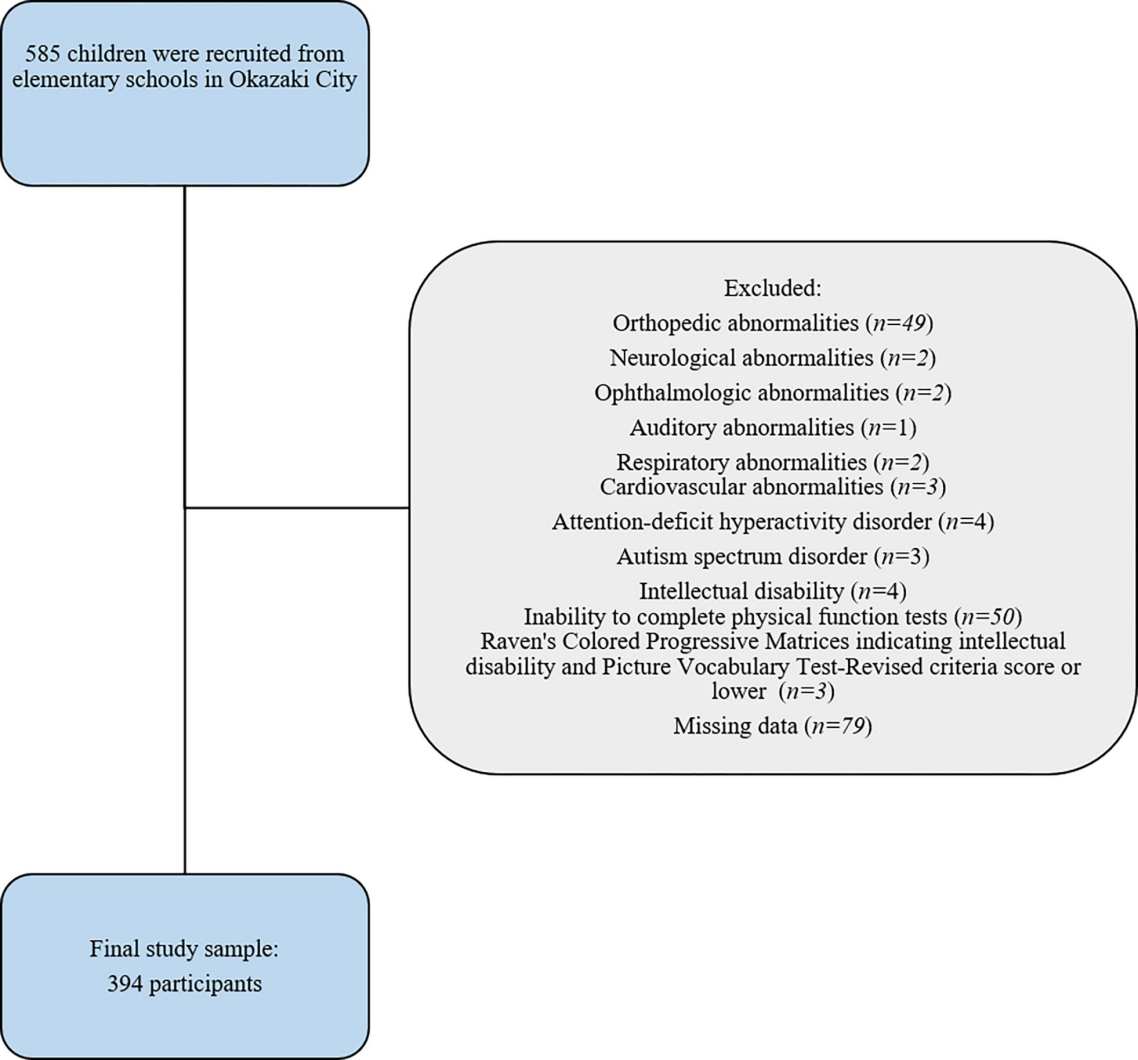
Flowchart of the enrollment of study participants.

Three of the 48 Okazaki municipal elementary schools were referred by the Okazaki Board of Education, and they provided informed consent to participate in this study. In total, 585 school-aged children (6–12 years) were recruited for this cross-sectional study between May 2018 and March 2023. Exclusion criteria included orthopedic (n = 49), neurological (n = 2), ophthalmologic (n = 2), auditory (n = 1), respiratory (n = 2), or cardiovascular abnormalities (n = 3) that could affect physical function test results; inability to complete physical function tests (n = 50); Raven’s Colored Progressive Matrices indicating intellectual disability and Picture Vocabulary Test-Revised [26, 27] criteria score or lower (n = 3); unable to perform a physical function assessment due to low-back pain (n = 0); and missing data (n = 79) (**Fig 1**). Of the 585 candidates, 191 were excluded, and 394 were enrolled in the study.

Low-back pain was assessed using a visual analog scale (0–10 cm). An orthopedic surgeon interviewed the participants to determine whether low-back pain occurred during movement [28]. Based on the pain severity evaluation by Boonstra et al., low-back pain severity was defined as ≥3 cm of symptoms in low-back pain [29]. Higher scores indicate more severe low-back pain. Based on this score, the participants were divided into children with low-back pain (score ≥3 cm) and those without low-back pain (score 0–2.9 cm).

This study was conducted in accordance with the Declaration of Helsinki, and the protocol was approved by the Ethics Committee of the Aichi Prefectural Mikawa Aoitori Ethics Review Board (approval number: 29002). The authors did not have access to information that could identify individual participants during or after data collection. The legal guardians of all participants provided written informed consent for their children to participate in the study and to release identifying information. All children consented to participate in the study.

### Data collection

#### Questionnaire

Moderate physical activity hours per week were assessed based on the Physical Activity Index recommended by the World Health Organization [30]. Children provided self-reported responses to the physical activity time questionnaire. Screen time was investigated by asking children to fill in the daily hours spent watching TV and movies. Their responses were verified by their parents. Parents and children rated sleep hours per day in relation to sleep history [30]. Furthermore, an orthopedic surgeon interviewed the participants to determine whether low-back pain occurred during movement. Low-back pain was assessed using a visual analog scale (0–10 cm). The participants were asked to perform trunk flexion and extension in a standing position to confirm low-back pain during movement [28]. In addition, for low-back pain, the patient was asked about the current pain level and when it began to manifest [28].

#### Measurement of the finger–floor distance

**Fig 2:**
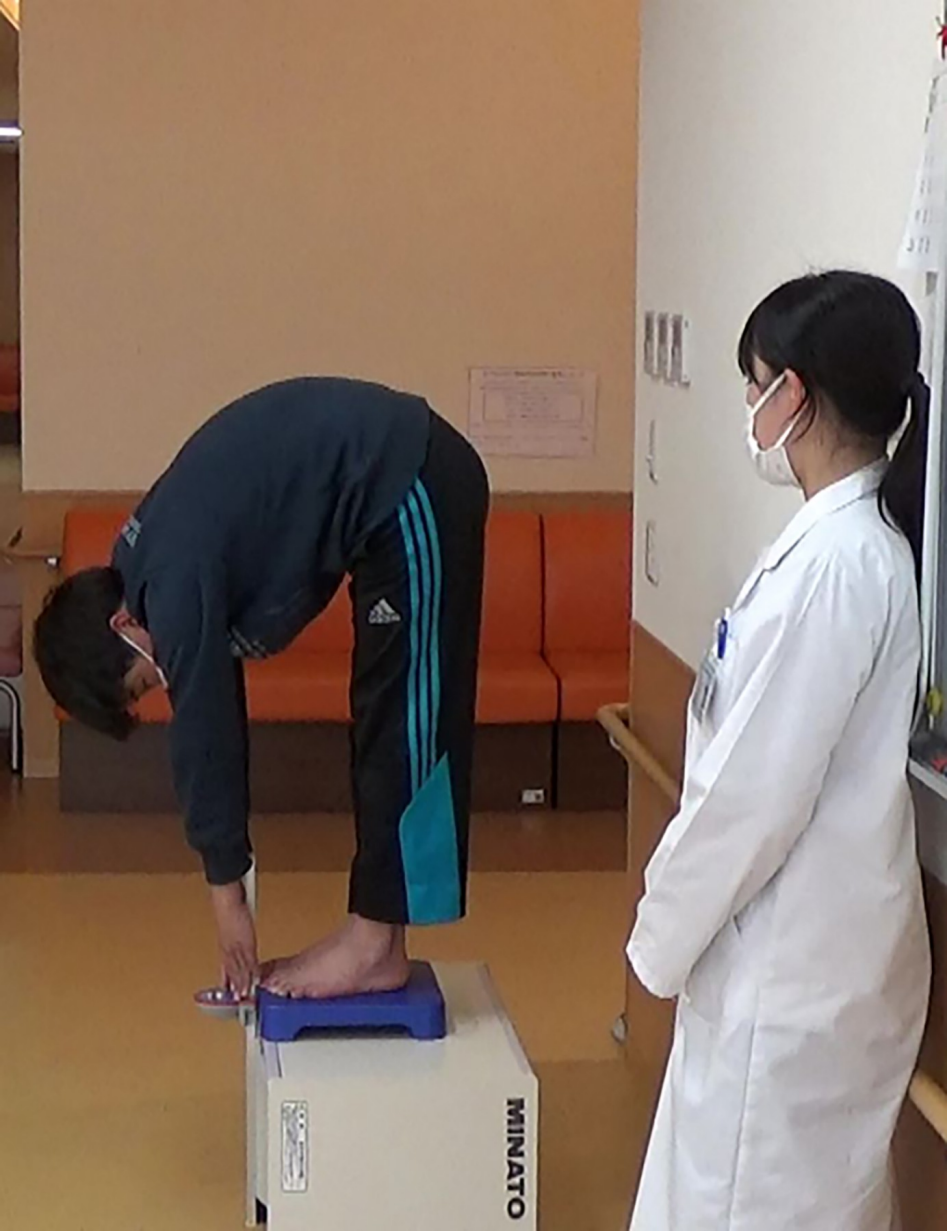
Measuring the fingertip-to-floor distance using a digital trunk forward flexion meter. If the fingertip did not reach the floor, the fingertip-to-floor distance was assigned a negative value; if it exceeded the distance to the floor, the distance was assigned a positive value.

The finger–floor distance (cm), a measure of flexibility, was evaluated using a digital trunk forward flexion meter (FLEXION-D; Takei Ltd., Niigata, Japan). The finger–floor distance was measured from the tips of the middle fingers to the floor in barefoot children with their knees straight and feet placed together and bent maximally forward **(Fig 2)** [31, 32]. When the tip of the middle finger reached the floor, 0 cm was indicated, and the absolute value increased with increasing distance from 0 cm. Negative values were obtained when the tip did not reach the floor. Measurements were recorded twice and in 0.1 cm increments, and the best result out of the two was considered the measured value. The shorter the finger–floor distance (fingertip not reaching the floor; negative value), the lower the flexibility. In a previous study, inter-rater reliability analysis for forward flexion measured by finger–floor distance had an acceptable intraclass correlation coefficient of 0.99 [33].

#### Back muscle strength

A digital back dynamometer (Back-D; Takei Ltd., Niigata, Japan) was used to measure back muscle strength [31, 34]. Participants were asked to stand on a platform with their feet shoulder-width apart. For adjustment by the research assistant, the chain length was adjusted according to the height difference. Back muscle strength was determined based on the maximum isometric extension force of the trunk muscles in the standing position, with the trunk flexed at 30° [31, 34]. This was undertaken to ensure that the force came from the back muscles, not the psoas or shoulder muscles, so the participant did not fall backward. Measurements were performed twice, and the average of the two values was recorded. Data on back muscle strength were corrected for weight. The reliability of the measurement tools evaluated by the intraclass correlation coefficient ranged from 0.93 to 0.97 [35].

### Pelvic tilt during gait

A physical therapist with more than 10 years of experience in clinical gait analysis performed the three-dimensional gait analysis using a motion analysis system (MX-T 20S; Vicon, Oxford, UK) with eight cameras and a sampling frequency of 100 Hz. The plug-in-gait model (May 2018 to March 2020) and Conventional Gait Model 2.3 (June 2020 to March 2023), with markers placed on the lower body, were used to measure gait [30, 36]. The children walked barefoot at a self-regulated speed on an 8-AMTI OPT force plate (Advanced Mechanical Technology, Inc., Watertown, MA, USA) in three trials [36]. Pelvic tilt was assessed using the maximum value in the sagittal plane during the stance phase. The mean maximum value of the pelvic tilt was calculated using the results of three gait trials analyzing the right and left lower legs [36].

### Sample size

The sample size was determined using G*Power (Heinrich Heine University, Düsseldorf, Germany) [37, 38]. Based on previous research, we considered the proportion of children with low-back pain to be 0.1 [4], with a statistical power of 0.8, two-tailed alphas of 0.05, and a medium effect size (d = 0.5). Based on these assumptions, the required sample size was 382 (35 children with low-back pain and a control group of 347).

### Statistical analysis

The normality of the distribution of each variable was checked using the Shapiro-Wilk test. The chi-squared test was used to compare the differences in the proportions of each sex in each group. Participant data are expressed as a mean (standard deviation) or median (range) and compared using an independent t-test or Mann–Whitney U test, as appropriate. Logistic regression was used to assess the association between finger–floor distance, back muscle strength, pelvic tilt during gait, and low-back pain. Statistical significance was defined as a two-sided p-value of <0.05. Multivariate logistic regression was used to determine the odds ratios of trunk function associated with low-back pain after controlling for sex as a confounding factor. The group (children with or without low-back pain) was the dependent variable in this analysis. All data analyses were performed using SPSS version 28.0 (IBM Corp., Armonk, NY, USA).

## Results

This study included 394 participants (191 boys and 203 girls; 38 with low-back pain and 356 without low-back pain) with a mean age of 8.9 (range, 6–12) years who were admitted for medical examination and physical function evaluation. All children with low-back pain had non-specific low-back pain, and 97.3% reported low-back pain during movement. Furthermore, none of the participants answered clearly regarding when the low-back pain began. **Tables 1 and 2** show the study participants’ demographic characteristics, trunk function measures, and a comparison between the two groups. Children with low-back pain were found to have reported a larger visual analog scale score (p < 0.0001; 95% confidence interval [CI]: -4.000 to -3.500) than those without low-back pain **(Table 1)**. Furthermore, there was a significant difference based on sex (p = 0.025; 95% CI: 1.091–4.440), with more boys reporting low-back pain (65.8%). There were no significant differences based on age, height, weight, BMI, physical activity time, screen time, or sleep time between the groups with and without low-back pain. Children with low-back pain had shorter finger–floor distances (p = 0.003; 95% CI: 1.183–5.586) than those without low-back pain **(Table 2)**. The two groups had no significant differences in back muscle strength or pelvic tilt during gait.

**Table 1:** Demographic characteristics of participants with and without low-back pain.

Variable	Children with low-back pain (n = 38)	Children without low-back pain (n = 356)	p-value	95% CI
Age (years), median (range)	9 (7–12)	9 (6–12)	0.182	-1.000–0.000
Sex, n (%)			0.025	1.091–4.440
Female	13 (34.2)	190 (53.4)		
Male	25 (65.8)	166 (46.6)		
Height (cm), median (range)	132.7 (110.7–158.7)	130.4 (107.5–173.2)	0.306	-6.400–1.900
Weight (kg), median (range)	27.6 (18.1–45.7)	27.2 (16.2–77.9)	0.407	-3.300–1.400
Body mass index (kg/m^2^), median (range)	15.9 (13.4–21.7)	15.8 (12.2–30.2)	0.940	-0.580–0.547
Physical activity (hours), median (range)	4.8 (0–21)	4.0 (0–22)	0.715	-1.000–1.500
Screen time per day (hours), median (range)	2.0 (0–5)	1.6 (0−9)	0.219	-0.500–0.000
Sleep time per day (hours), median (range)	9 (6–10)	9 (6.5–11)	0.415	0.000–0.000
Visual analog scale (cm), median (range)	4 (3–10)	0 (0–2)	0.0001	-4.000 –-3.500

The p-value for the difference in the proportion by sex was calculated using the chi-squared test, and the other p-values were calculated using the Mann–Whitney U test.

CI, confidence interval

**Table 2:** Physical performance of the trunk in participants with and without low-back pain.

Variable	Children with low-back pain (n = 38)	Children without low-back pain (n = 356)	p-value	95% CI
Fingertip-to-floor distance (cm)	1.9 (7.2)	5.2 (6.5)	0.003	1.183–5.586
Back muscle strength (kg/kg)	1.2 (0.6−2.0)	1.1 (0.6−2.2)	0.287	-4.750–1.500
Pelvic tilt during gait (degree)	15.5 (4.3)	15.6 (4.3)	0.859	-1.307–1.568

Data are presented as a mean (standard deviation) or median (range).

The p-values of the fingertip-to-floor distance and pelvic tilt were calculated using the independent t-test, and the other p-values were calculated using the Mann–Whitney U test.

CI, confidence interval

Multivariate logistic regression analyses for low-back pain, performed among trunk functions, showed that the finger–floor distance (odds ratio 0.921; P = 0.007; 95% CI: 0.868–0.977) was associated with low-back pain. In contrast, back muscle strength and pelvic tilt during walking were not associated with low-back pain (**Table 3**).

**Table 3:** Relationship between the physical performance of the trunk and low-back pain.

Variable	β	SE	Wald	Odds ratio (95% CI)	p-value
Fingertip-to-floor distance	-0.082	0.030	7.345	0.921 (0.868−0.977)	0.007
Back muscle strength	0.919	0.641	2.052	2.506 (0.713−8.810)	0.152
Pelvic tilt during gait	0.034	0.043	0.620	1.035 (0.951−1.126)	0.431
Sex	0.442	0.395	1.247	1.555 (0.717−3.375)	0.264

Thirty-eight children with and 356 without low-back pain were analyzed. The occurrence of low-back pain was the dependent variable (without low-back pain = 0, with low-back pain = 1), and sex was an adjustment variable.

β, partial regression coefficient; CI, confidence interval; SE, standard error.

## Discussion

The current study aimed to examine the association between flexibility and low-back pain in children. The key finding was that flexibility was associated with low-back pain. In this cross-sectional study, using the odds ratio of a multivariable logistic regression, we observed that each additional shorter finger–floor distance was related to a higher frequency of reporting low-back pain.

Previous studies reported decreased flexibility of the hamstring and quadriceps muscles in adolescents with low-back pain [21, 39]. Furthermore, stiffness of the thoracolumbar fascia results from rapid growth and the development of low-back pain due to preexisting inflexibility [40]. However, another study reported that flexibility and muscle strength were not associated with low-back pain [41]. The studies analyzing the relationship between low-back pain and flexibility in children are lacking, and further investigation, including longitudinal studies, is warranted. The finger–floor distance was shorter in children with low-back pain than in those without low-back pain. The results of the current study showed no differences in age between groups, suggesting that low flexibility is associated with low-back pain in 6–12-year-old children. Thus, poor flexibility of the trunk and lower extremities may be related to low-back pain in the pediatric population. Moreover, the differences in these results may be due to differences in race and lifestyle.

Several reports have demonstrated that low-back pain in children is as frequent a complaint as in adults [3, 4, 42]. In another study, the incidence of low-back pain in children was 10.2% [4], which was similar to the 9.6% result in our study. Furthermore, low-back pain during movement accounted for 97.3% of all non-specific low-back pain, indicating that the cause of the apparent low-back pain is unknown and that evaluation of back pain in children must be done with caution. This term is used when the pathoanatomical cause of pain cannot be identified [43]. Although detailed reports on low-back pain in Japanese elementary school children are scarce, and no definite conclusions may be drawn, our results suggest that low-back pain in Japanese children is more common in boys than in girls. Hence, boys may be exposed to potentially more dangerous and strenuous sporting activities than girls. However, logistic regression analysis did not show an association between sex and low-back pain. Nevertheless, a Spanish study has reported a higher rate of back pain in girls than in boys [9]. Meanwhile, there are reports of an unclear relationship between low-back pain and sex [44]. The differences in these results may be related to lifestyle and exercise habits in different countries [45, 46]. There may also be differences based on the target age group, though these differences have not yet been clarified and require further investigation.

Back muscle strength was not a risk factor for low-back pain in this study, suggesting that weak back muscle strength is not necessarily related to low-back pain. A recent study reported that trunk muscle strength was not associated with low-back pain in children, although extensor muscle endurance was associated with low-back pain [41]. Noll et al. reported that trunk extensor muscle endurance deficits might increase vulnerability to tissue strain during sporting activities [41]. However, present study evaluated back muscle strength rather than endurance, which is a topic for future research.

This study found no association between low-back pain and pelvic tilt during walking. This result was consistent with that of other studies showing a lack of a relationship between pelvic tilt and low-back pain in children [47, 48].

BMI, physical activity, screen time, and sleep time were not associated with low-back pain in the present study’s participants. These results were inconsistent with those of previous studies [13, 14]. Low-back pain did not affect these factors in this study, indicating that children with low-back pain do not necessarily exhibit greater changes in BMI, physical activity, screen time, and sleep time. Moreover, it is necessary to consider that the participants’ age range and inclusion and exclusion criteria can influence the results.

This study had a few limitations. First, this cross-sectional study only demonstrated the association between flexibility or physical performance of the trunk and low-back pain; therefore, it is impossible to prove an apparent causal association. Second, the low-back pain assessments were based on self-reported data. Third, the study sample was relatively small and included only community-dwelling school-aged children from Japan. However, the results of this study could be used in elementary schools in the community to promote education on low-back pain prevention.

Despite these limitations, the present study had several strengths. This was a cross-sectional study to test the hypothesis that low-back pain in childhood is associated with decreased flexibility. Furthermore, this was the first study in Japan conducted among elementary school children to demonstrate an association between low-back pain and flexibility in childhood, which has not been clearly understood as a distinct characteristic compared with adults and older adults. In addition, study participants were assessed for flexibility using a physical function assessment that could be adopted and reproduced in a clinical setting.

In the present study, the prevalence of low-back pain in children was 9.6%, of which 97.3% had low-back pain during movement, and boys were more likely to have low-back pain. Low-back pain was only associated with flexibility. Back muscle strength, pelvic tilt during gait, and sex were not associated with low-back pain. Therefore, it is important to consider that children who experience low-back pain during exercise are more likely to have reduced flexibility. These findings have implications for preventing low-back pain in children and may provide useful information for clinicians and researchers. However, given our findings, future studies with larger sample sizes are required to further investigate this issue. If our findings are confirmed in future studies, clinicians, patients, and researchers may consider flexibility in the assessment of low-back pain. At this time, it may be clinically relevant for clinicians and researchers to inform patients that decreased flexibility is a contributing factor to low-back pain. This is because there are rehabilitation options that can help address the symptoms that lead to loss of flexibility.

## Conclusions

The present study demonstrated that the risk for low-back pain was associated with flexibility, regardless of sex and muscle strength, with a shorter finger–floor distance found in children with low-back pain. It indicated that low-back pain is common in boys (65.8%), with an entirety of 9.6%. Improved knowledge and awareness of the risk factors for low-back pain and the need for good flexibility are areas that require further investigation. Interventions to improve flexibility can reduce the degree of pain experienced by children with low-back pain.

## Supporting information

S1 File (XLSX)

## References

[1] Hangai M, Kaneoka K, Okubo Y, Miyakawa S, Hinotsu S, Mukai N, et al. Relationship between low back pain and competitive sports activities during youth. Am J Sports Med. 2010;38: 791–796. doi: 10.1177/0363546509350297 20051500

[2] Maher C, Underwood M, Buchbinder R. Non-specific low back pain. Lancet. 2017;389: 736–747. doi: 10.1016/S0140-6736(16)30970-9 27745712

[3] Taimela S, Kujala UM, Salminen JJ, Viljanen T. The prevalence of low back pain among children and adolescents. A nationwide, cohort-based questionnaire survey in Finland. Spine (Phila Pa 1976). 1997;22: 1132–1136. doi: 10.1097/00007632-199705150-00013 9160472

[4] Sato T, Ito T, Hirano T, Morita O, Kikuchi R, Endo N, et al. Low back pain in childhood and adolescence: A cross-sectional study in Niigata City. Eur Spine J. 2008;17: 1441–1447. doi: 10.1007/s00586-008-0788-5 18830637 PMC2583196

[5] Balagué F, Troussier B, Salminen JJ. Non-specific low back pain in children and adolescents: risk factors. Eur Spine J. 1999;8: 429–438. doi: 10.1007/s005860050201 10664299 PMC3611213

[6] Junge T, Wedderkopp N, Boyle E, Kjaer P. The natural course of low back pain from childhood to young adulthood–A systematic review. Chiropr Man Therap. 2019;27: 10. doi: 10.1186/s12998-018-0231-x 30931103 PMC6425623

[7] Ben Ayed H, Yaich S, Trigui M, Ben Hmida M, Ben Jemaa M, Ammar A, et al. Prevalence, risk factors and outcomes of neck, shoulders and low-back pain in secondary-school children. J Res Health Sci. 2019;19: e00440. doi: 10.15171/jrhs.2019.07 31133629 PMC6941626

[8] Alsiddiky A, Alatassi R, Alsaadouni FN, Bakerman K, Awwad W, Alenazi A, et al. Assessment of perceptions, knowledge, and attitudes of parents regarding children’s schoolbags and related musculoskeletal health. J Orthop Surg Res. 2019;14: 113. doi: 10.1186/s13018-019-1142-9 31029176 PMC6487022

[9] Martínez-Romero MT, Cejudo A, Sainz de Baranda P. Prevalence and characteristics of back pain in children and adolescents from the region of Murcia (Spain): ISQUIOS programme. Int J Environ Res Public Health. 2022;19: 946. doi: 10.3390/ijerph19020946 35055768 PMC8775889

[10] Lardon A, Leboeuf-Yde C, Le Scanff C, Wedderkopp N. Is puberty a risk factor for back pain in the young? A systematic critical literature review. Chiropr Man Therap. 2014;22: 27. doi: 10.1186/s12998-014-0027-6 25328668 PMC4200222

[11] Kamper SJ, Williams CM. Musculoskeletal pain in children and adolescents: A way forward. J Orthop Sports Phys Ther. 2017;47: 702–704. doi: 10.2519/jospt.2017.0109 28967337

[12] Hebert JJ, Leboeuf-Yde C, Franz C, Lardon A, Hestbæk L, Manson N, et al. Pubertal development and growth are prospectively associated with spinal pain in young people (CHAMPS study-DK). Eur Spine J. 2019;28: 1565–1571. doi: 10.1007/s00586-019-05905-6 30740638

[13] Beynon AM, Hebert JJ, Lebouef-Yde C, Walker BF. Potential risk factors and triggers for back pain in children and young adults. A scoping review, part II: Unclear or mixed types of back pain. Chiropr Man Therap. 2019;27: 61. doi: 10.1186/s12998-019-0281-8 31827768 PMC6862810

[14] Pate JW, Joslin R, Hurtubise K, Anderson DB. Assessing a child or adolescent with low back pain is different to assessing an adult with low back pain. J Paediatr Child Health. 2022;58: 566–571. doi: 10.1111/jpc.15933 35218582 PMC9303678

[15] Nagai T, Abt JP, Sell TC, Keenan KA, Clark NC, Smalley BW, et al. Lumbar spine and hip flexibility and trunk strength in helicopter pilots with and without low back pain history. Work. 2015;52: 715–722. doi: 10.3233/WOR-152192 26528848

[16] Sung PS. A kinematic analysis for shoulder and pelvis coordination during axial trunk rotation in subjects with and without recurrent low back pain. Gait Posture. 2014;40: 493–498. doi: 10.1016/j.gaitpost.2014.06.001 25008865

[17] Radwan A, Bigney KA, Buonomo HN, Jarmak MW, Moats SM, Ross JK, et al. Evaluation of intra-subject difference in hamstring flexibility in patients with low back pain: an exploratory study. J Back Musculoskelet Rehabil. 2014. doi: 10.3233/BMR-140490 24968796

[18] Lloyd RS, Oliver JL, Faigenbaum AD, Myer GD, De Ste Croix MB. Chronological age vs. biological maturation: implications for exercise programming in youth. J Strength Cond Res. 2014;28: 1454–1464. doi: 10.1519/JSC.0000000000000391 24476778

[19] Salminen JJ, Maki P, Oksanen A, Pentti J. Spinal mobility and trunk muscle strength in 15-year-old schoolchildren with and without low-back pain. Spine (Phila Pa 1976). 1992;17: 405–411. doi: 10.1097/00007632-199204000-00005 1533731

[20] Hultman G, Saraste H, Ohlsen H. Anthropometry, spinal canal width, and flexibility of the spine and hamstring muscles in 45-55-year-old men with and without low back pain. J Spinal Disord. 1992;5: 245–253. doi: 10.1097/00002517-199209000-00001 1387820

[21] Feldman DE, Shrier I, Rossignol M, Abenhaim L. Risk factors for the development of low back pain in adolescence. Am J Epidemiol. 2001;154: 30–36. doi: 10.1093/aje/154.1.30 11427402

[22] Balagué F, Damidot P, Nordin M, Parnianpour M, Waldburger M. Cross-sectional study of the isokinetic muscle trunk strength among school children. Spine (Phila Pa 1976). 1993;18: 1199–1205. doi: 10.1097/00007632-199307000-00013 8362327

[23] Achar S, Yamanaka J. Back pain in children and adolescents. Am Fam Physician. 2020;102: 19–28. 32603067

[24] García-Moreno JM, Calvo-Muñoz I, Gómez-Conesa A, López-López JA. Effectiveness of physiotherapy interventions for back care and the prevention of non-specific low back pain in children and adolescents: a systematic review and meta-analysis. BMC Musculoskelet Disord. 2022;23: 314. doi: 10.1186/s12891-022-05270-4 35366847 PMC8976404

[25] Noll M, Wedderkopp N, Mendonça CR, Kjaer P. Motor performance and back pain in children and adolescents: a systematic review and meta-analysis protocol. Syst Rev. 2020;9: 212. doi: 10.1186/s13643-020-01468-6 32928303 PMC7491087

[26] Raven J, Raven JC, Court JH. Manual for Raven’s progressive matrices and vocabulary scales. Oxford, UK: Oxford Psychologists Press; 1998.

[27] Ueno K, Nagoshi N, Konuki S. Picture vocabulary test revised. [Nihon Bunka kagakusha]. Tokyo, Japan; 2008.

[28] Gu Y, Ito T, Ito Y, Noritake K, Ochi N, Matsunaga N, et al. Factors related to locomotive syndrome in school-aged children in Okazaki: A cross-sectional study. Healthcare (Basel). 2021;9: 1595. doi: 10.3390/healthcare9111595 34828640 PMC8619500

[29] Boonstra AM, Schiphorst Preuper HR, Balk GA, Stewart RE. Cut-off points for mild, moderate, and severe pain on the visual analogue scale for pain in patients with chronic musculoskeletal pain. Pain. 2014;155: 2545–2550. doi: 10.1016/j.pain.2014.09.014 25239073

[30] Ito T, Sugiura H, Ito Y, Narahara S, Noritake K, Takahashi D, et al. Physical functions among children before and during the COVID-19 pandemic: A prospective longitudinal observational study (Stage 1). Int J Environ Res Public Health. 2022;19: 11513. doi: 10.3390/ijerph191811513 36141790 PMC9517518

[31] Kwon HJ, Kim YL, Lee HS, Lee SM. A study on the physical fitness of children with juvenile rheumatoid arthritis. J Phys Ther Sci. 2017;29: 378–383. doi: 10.1589/jpts.29.378 28356614 PMC5360993

[32] Uehara M, Takahashi J, Ikegami S, Kuraishi S, Futatsugi T, Oba H, et al. Correlation of lower instrumented vertebra with spinal mobility and health-related quality of life after posterior spinal fusion for adolescent idiopathic scoliosis. Clin Spine Surg. 2019;32: E326–E329. doi: 10.1097/BSD.0000000000000794 31361270

[33] Perret C, Poiraudeau S, Fermanian J, Colau MM, Benhamou MA, Revel M. Validity, reliability, and responsiveness of the fingertip-to-floor test. Arch Phys Med Rehabil. 2001;82: 1566–1570. doi: 10.1053/apmr.2001.26064 11689977

[34] Gu Y, Ito T, Ito Y, Noritake K, Ochi N, Matsunaga N, et al. Physical activity related to body muscle mass index and stiffness index in 7-to-10-Year-Old girls. Healthcare (Basel). 2022;10: 197. doi: 10.3390/healthcare10020197 35206812 PMC8871963

[35] Essendrop M, Bente S, Klaus H. Reliability of isometric muscle strength tests for the trunk, hands and shoulders. Int J Ind Ergon. 2001;28: 379–387. doi: 10.1016/S0169-8141(01)00044-0

[36] Ito T, Noritake K, Ito Y, Tomita H, Mizusawa J, Sugiura H, et al. Three-dimensional gait analysis of lower extremity gait parameters in Japanese children aged 6 to 12 years. Sci Rep. 2022;12: 7822. doi: 10.1038/s41598-022-11906-1 35551257 PMC9098504

[37] Faul F, Erdfelder E, Lang AG, Buchner A. G*Power 3: A flexible statistical power analysis program for the social, behavioral, and biomedical sciences. Behav Res Methods. 2007;39: 175–191. doi: 10.3758/bf03193146 17695343

[38] Faul F, Erdfelder E, Buchner A, Lang AG. Statistical power analyses using G*Power 3.1: tests for correlation and regression analyses. Behav Res Methods. 2009;41: 1149–1160. doi: 10.3758/BRM.41.4.1149 19897823

[39] Kanchanomai S, Janwantanakul P, Pensri P, Jiamjarasrangsi W. A prospective study of incidence and risk factors for the onset and persistence of low back pain in Thai university students. Asia Pac J Public Health. 2015;27: NP106-NP115. doi: 10.1177/1010539511427579 22186386

[40] MacDonald J, Stuart E, Rodenberg R. Musculoskeletal low back pain in school-aged children: a review. JAMA Pediatr. 2017;171: 280–287. doi: 10.1001/jamapediatrics.2016.3334 28135365

[41] Noll M, Kjaer P, Mendonça CR, Wedderkopp N. Motor performance and back pain in children and adolescents: A systematic review. Eur J Pain. 2022;26: 77–102. doi: 10.1002/ejp.1850 34365693

[42] Watson KD, Papageorgiou AC, Jones GT, Taylor S, Symmons DP, Silman AJ, et al. Low back pain in schoolchildren: occurrence and characteristics. Pain. 2002;97: 87–92. doi: 10.1016/s0304-3959(02)00008-8 12031782

[43] Miñana-Signes V, Monfort-Pañego M, Bosh-Bivià AH, Noll M. Prevalence of low back pain among primary school students from the City of Valencia (Spain). Healthcare (Basel). 2021;9: 270. doi: 10.3390/healthcare9030270 33802400 PMC8000525

[44] Huguet A, Tougas ME, Hayden J, McGrath PJ, Stinson JN, Chambers CT. Systematic review with meta-analysis of childhood and adolescent risk and prognostic factors for musculoskeletal pain. Pain. 2016;157: 2640–2656. doi: 10.1097/j.pain.0000000000000685 27525834

[45] Sanders SH, Brena SF, Spier CJ, Beltrutti D, McConnell H, Quintero O. Chronic low back pain patients around the world: cross-cultural similarities and differences. Clin J Pain. 1992;8: 317–323. doi: 10.1097/00002508-199212000-00006 1493342

[46] Calvo-Muñoz I, Kovacs FM, Roqué M, Gago Fernández I, Seco Calvo J. Risk factors for low back pain in childhood and adolescence: A systematic review. Clin J Pain. 2018;34: 468–484. doi: 10.1097/AJP.0000000000000558 28915154

[47] Nourbakhsh MR, Arab AM. Relationship between mechanical factors and incidence of low back pain. J Orthop Sports Phys Ther. 2002;32: 447–460. doi: 10.2519/jospt.2002.32.9.447 12322811

[48] Day JW, Smidt GL, Lehmann T. Effect of pelvic tilt on standing posture. Phys Ther. 1984;64: 510–516. doi: 10.1093/ptj/64.4.510 6231648

